# Rapid On-Site Detection of Extensively Drug-Resistant Genes in *Enterobacteriaceae* via Enhanced Recombinase Polymerase Amplification and Lateral Flow Biosensor

**DOI:** 10.1128/spectrum.03344-22

**Published:** 2022-11-29

**Authors:** Jin Tao, Dejun Liu, Jincheng Xiong, Leina Dou, Weishuai Zhai, Rong Zhang, Yang Wang, Jianzhong Shen, Kai Wen

**Affiliations:** a Beijing Advanced Innovation Center for Food Nutrition and Human Health, College of Veterinary Medicine, China Agricultural University, Beijing, People’s Republic of China; b Clinical Microbiology Laboratory, 2nd Affiliated Hospital of Zhejiang University, School of Medicine, Zhejiang University, Hangzhou, People’s Republic of China; Yangzhou University

**Keywords:** multi-drug resistance genes, isothermal amplification, point-of-care testing, cascade detection system, colorimetric detection

## Abstract

The widespread emergence of transferable extensively drug-resistant (XDR) genes, including *bla*_NDM_ and *bla*_KPC_ for carbapenem resistance, *mcr-1* for colistin resistance, and *tet*(X4) and *tet*(X6) for tigecycline resistance, in *Enterobacteriaceae* poses a major threat to public health. Thus, rapid on-site detection of these XDR genes is urgently needed. We developed a cascade system with a unitary polyethylene glycol (PEG) 200-enhanced recombinase polymerase amplification (RPA) as the core, combined with a modified Chelex-100 lysis method and a horseradish peroxidase (HRP)-catalyzed lateral flow immunoassay (LFIA) biosensor, to accurately detect these genes in *Enterobacteriaceae*. The conventional Chelex-100 lysis method was modified to allow *in situ* extraction of bacterial DNA in 20 min without requiring bulky high-speed centrifuges. Using PEG 200 increased the amplification efficiency of the RPA by 13%, and the HRP-catalyzed LFIA biosensor intensified the colorimetric signal of the test line. Following optimization, the sensitivity of the cascade system was <10 copies/μL with satisfactory specificity, allowing for highly sensitive detection of these XDR genes in *Enterobacteriaceae*. The complete detection procedure can be completed in less than 1 h without using large-scale instruments. This assay is conducive to rapid on-site visual detection of these XDR genes in *Enterobacteriaceae* in practical applications, thus providing better technical support for clinical surveillance of these genes and better treatment of XDR pathogens.

**IMPORTANCE** Carbapenem, colistin, and tigecycline are considered the last resorts for treating severe bacterial infections caused by extensively drug-resistant (XDR) pathogens. A major threat to public health is the emergence and prevalence of transferable XDR genes in *Enterobacteriaceae*, such as *bla*_NDM_ and *bla*_KPC_ for carbapenem resistance, *mcr-1* for colistin resistance, and *tet*(X4) and *tet*(X6) for tigecycline resistance. Therefore, it is imperative to develop rapid on-site methods to detect these XDR genes. In this study, we constructed a cascade system for detecting these genes based on PEG 200-enhanced recombinase polymerase amplification combined with a modified Chelex-100 lysis method and HRP-catalyzed lateral flow immunoassay. The current method is capable of detecting the above-mentioned XDR genes *in situ* with satisfactory specificity and sensitivity, which could provide technical support for the surveillance of these genes and provide medication recommendations for the treatment of relevant clinical infections.

## INTRODUCTION

Antimicrobial resistance is a global threat to human health and to treatment of bacterial infections. Carbapenem, colistin, and tigecycline are last-resort antibiotics for treating serious infections caused by extensively drug-resistant (XDR) bacteria (1–3). However, resistance among clinical bacterial pathogens to these critically important antibiotics is increasing and has severely affected the clinical use of these drugs. Both metallo-β-lactamase NDM and serine β-lactamase KPC cause carbapenem-resistant *Enterobacteriaceae* (CRE) infections, but because they have different susceptibilities to certain antibiotics (e.g., aztreonam), the two carbapenemases must be clearly distinguished before treatment to prevent antibiotic misuse (4). Recent identification of the plasmid-mediated transferable colistin resistance gene *mcr-1* and the tigecycline resistance genes *tet*(X4) and *tet*(X6) in *Enterobacteriaceae* has narrowed the options for treating CRE infections and has impacted the clinical use of colistin and tigecycline (5, 6). Meanwhile, *bla*_NDM_/*bla*_KPC_, *mcr-1*, and *tet*(X4)/*tet*(X6) are the most prevalent carbapenem, colistin, and tigecycline resistance genes in *Enterobacteriaceae*, respectively (7, 8). Therefore, fast detection of these resistance genes is crucial to better diagnose infections and administer suitable antimicrobial agents. PCR-based assays are the most commonly used methods to detect these XDR genes; however, such methods require a sophisticated and specialized thermal cycler to operate and 3 to 4 h to obtain results (9). This can cause delays in clinical treatment and lead to inappropriate antibiotic use. Hence, developing rapid on-site tests to determine clinical XDR genes is necessary for fast infection diagnosis and appropriate antibiotic prescribing.

To rapidly detect pathogens in the field, clinicians are applying isothermal amplification techniques that do not require complex thermal cyclers and can rapidly amplify target genes at constant temperatures. Innovative technologies in this field include rolling circle amplification, loop-mediated isothermal amplification (LAMP), and recombinase polymerase amplification (RPA) (10, 11). Of these, RPA is highly sensitive and can complete the nucleic acid amplification process within 30 min (12, 13). RPA involves a nucleic acid amplification reaction mixture consisting of recombinase, polymerase, and single-stranded binding protein with crowding reagents in the range of 37°C to 43°C based on strand exchange reactions (14).

However, achieving rapid *in situ* detection of these XDR genes requires bacterial DNA extraction and subsequent detection of the nucleic acid amplification product. The current procedures for most commercially available nucleic acid extraction kits used to extract bacterial DNA often take over an hour to complete and require bulky high-speed centrifuges, which do not allow quick and easy detection (15). Therefore, a rapid bacterial DNA extraction method suitable for field use is needed. To achieve rapid on-site detection of RPA products, we previously proposed the lateral flow immunoassay (LFIA), a paper-based platform that takes advantage of antigen-antibody-specific reactions and nanomaterial-labeling techniques for qualitative target detection (16).

Here, we constructed a cascade detection system using enhanced RPA as the core, along with a modified bacterial DNA extraction method and a horseradish peroxidase (HRP)-catalyzed LFIA biosensor. The proposed assay enables highly sensitive and specific on-site detection of *bla*_NDM_, *bla*_KPC_, *mcr-1*, *tet*(X4), and *tet*(X6) within 1 h without relying on specialized equipment, thus providing a better monitoring tool for preventing and controlling these XDR genes, as well as a suitable reference for guiding clinical antibiotic use.

## RESULTS AND DISCUSSION

### Principles.

**FIG 1:**
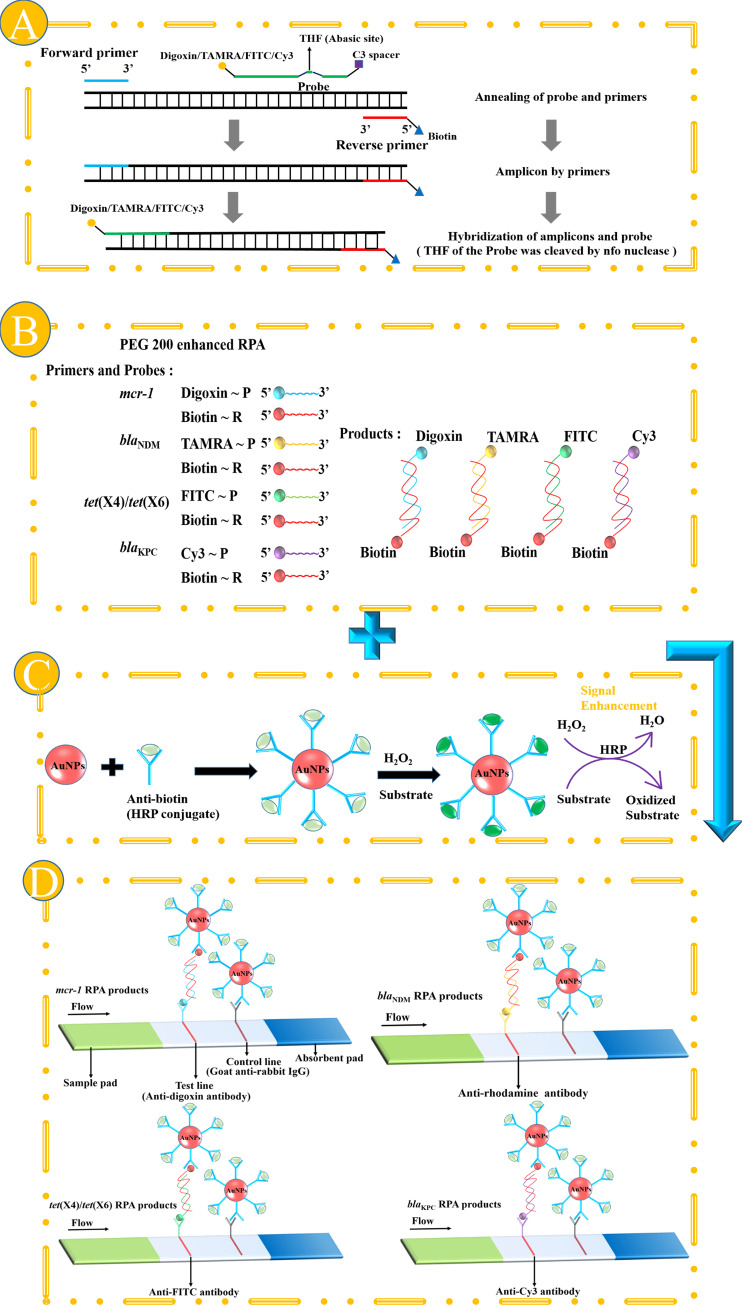
Schematic diagram of the principle and design of nfo-RPA, unitary PEG 200-enhanced RPA, and HRP-catalyzed enzymatic reaction-enhanced lateral flow biosensor. (A) nfo-RPA; (B) unitary PEG 200-enhanced RPA; (C) AuNP-antibody (HRP conjugate) conjugation; (D) HRP-catalyzed enzymatic reaction-enhanced lateral flow biosensor.

**(i) Principle of the nfo-RPA.** The nfo-RPA reverse primers were labeled with biotin, and the probes were labeled with digoxin/carboxytetramethylrhodamine (TAMRA)/fluorescein isothiocyanate (FITC)/Cy3 (Fig. 1A). Amplicons generated by forward/reverse primers were hybridized with probes to produce amplicons formed by the probe/reverse primers. The nfo-RPA enabled production of numerous biotin- and digoxin/TAMRA/FITC/Cy3-linked double-stranded DNAs, which manifested the presence of XDR genes (Fig. 1B). Four sets of primers and probes for nfo-RPA were designed and validated using HRP-catalyzed enzymatic reaction-enhanced LFIA (see Fig. S2 in the supplemental material).

**(ii) HRP-AuNP-antibody conjugation.** The HRP-gold nanoparticle (AuNP)-antibody conjugate was fabricated as the signal component in the constructed LFIA system (Fig. 1C). HRP catalyzes the reaction between H_2_O_2_ and AEC (3,3′,5,5′-3-amino-9-ethylcarbazole) to generate chromatic peroxidase products. AEC and H_2_O_2_ were then applied to the nitrocellulose (NC) membrane after the LFIA. The colored products yielded by the HRP-catalyzed enzymatic reaction are deposited on the surfaces of the AuNPs to enhance the optical effect and the test and control line intensities (17).

**(iii) HRP-catalyzed LFIA biosensor.** The double-stranded DNA complexes generated by amplification were detected using the HRP-catalyzed LFIA biosensor (Fig. 1D). The rationale of the fundamental LFIA measurement is based on sandwich-type “(antibiotin-AuNPs)-(target DNA)-(associated antibody)” hybridization reactions. This HRP-enhanced LFIA improved this version of the layout. The HRP-catalyzed enzymatic reaction significantly increased the signal intensity of the LFIA test line from 366.44 to 423.77 (*P* < 0.01, Fig. S3). The presence or absence of a red stripe on the test line indicates an XDR gene positive or XDR gene negative result, respectively. Control lines demonstrated that the biosensor operated well.

### Modified Chelex-100 lysis method for bacterial DNA extraction in 20 min without centrifugation.

**FIG 2:**
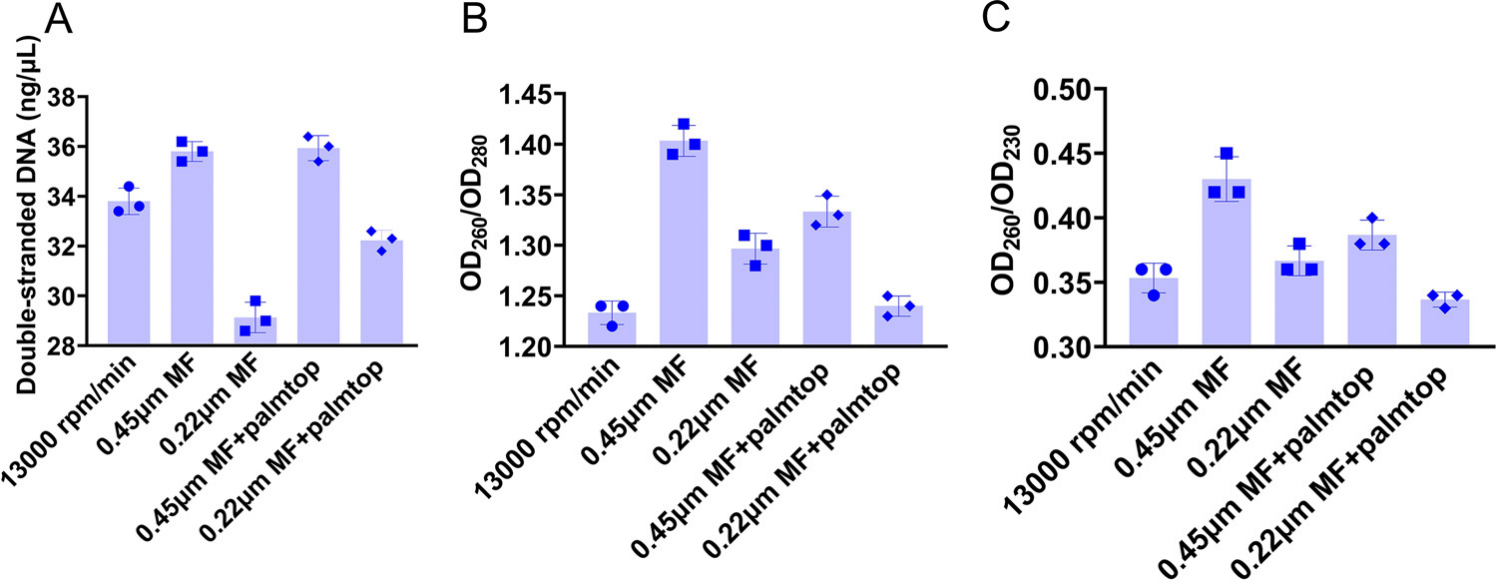
Modification of the conventional Chelex-100 lysis method. (A) Concentration of the double-stranded DNA; (B) OD_260_/OD_280_ value; (C) OD_260_/OD_230_ value. MF, membrane filtration; palmtop, palmtop centrifugation.

Bacterial DNA extraction is an important preprocessing step in detecting XDR genes. However, the most commonly used commercial kit extraction method requires a high-speed centrifuge, and the entire process takes over 1 h. Chelex-100 is a chemical chelating resin that enables bacterial lysis and DNA release under boiling conditions (18, 19). This process can be completed in 30 min but requires a high-speed centrifuge. To construct a rapid on-site assay for XDR genes, we attempted to optimize this method by using membrane filtration rather than centrifugation. Compared with high-speed centrifugation, the 0.45-μm filter membrane increased the concentration from 33.8 ng/μL to 35.8 ng/μL and the optical density at 260 nm (OD_260_)/OD_280_ of the extracted bacterial DNA from 1.23 to 1.40 (Fig. 2). The Chelex-100 residue, which cannot be eliminated by high-speed centrifugation, can chelate and inhibit downstream nucleic acid amplification reactions (20). No residual Chelex-100 was detected at the bottom of the tubes after filtration with a 0.45-μm filter membrane; therefore, inhibition of the nucleic acid amplification by residual Chelex-100 was minimized (Fig. S4). This modified method was further validated using the constructed standard strains. Using the standard *mcr-1*-carrying strain as an example, compared with the commercial kit, the modified Chelex-100 lysis method reduced the concentration of the extracted DNA from 47.4 to 27.7 ng/μL and the OD_260_/OD_280_ from 2.02 to 1.42 (Fig. S5). Although both the concentration and quality of the extracted bacterial DNA were reduced compared to those with the commercial bacterial DNA extraction kit, our modified Chelex-100 lysis method significantly decreased the extraction time of bacterial DNA to less than 20 min while maintaining the assay’s specificity and sensitivity. The modified method also enabled extraction of bacterial DNA from 95 strains from environmental and clinical collections and met the requirements for subsequent nucleic acid amplification (Table S2). The optimized Chelex-100 lysis method enabled DNA extraction in 20 min without requiring a high-speed centrifuge, thus making it more suitable for field applications.

### One percent PEG 200 increased the amplification efficiency of RPA by 13%.

**FIG 3:**
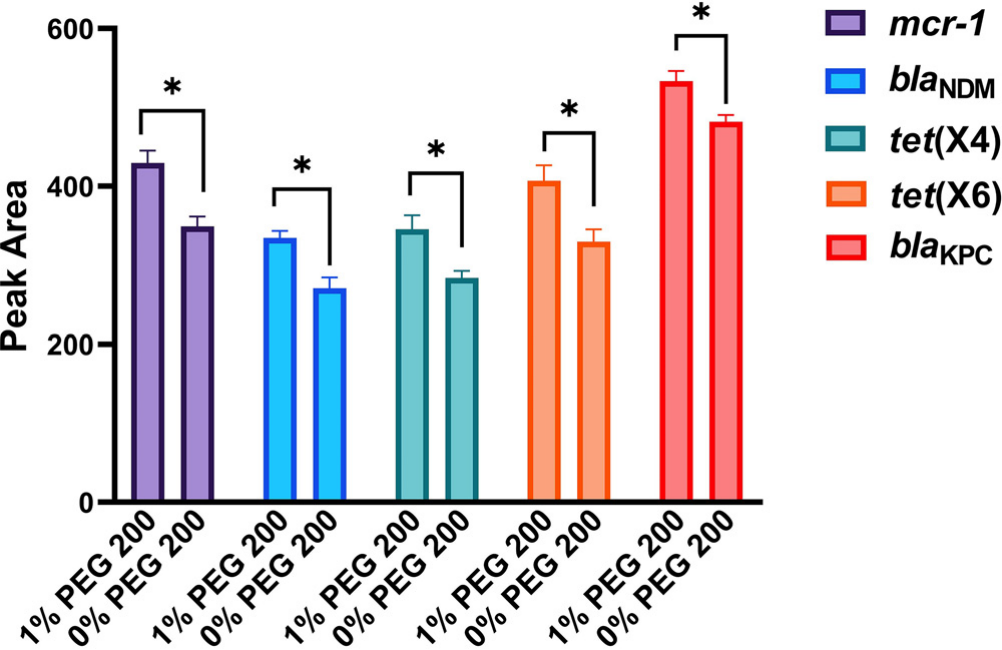
Validation of the improved RPA amplification efficiency of PEG 200 at optimized working concentrations for different XDR genes using the constructed HRP-catalyzed LFIA biosensor.

Polyethylene glycol (PEG) 200 is a kind of polyethylene glycol with a molecular weight of 200. PEG 200 improved PCR amplification efficiency by stabilizing the primer-template complex formed during PCR, indicating the importance of PEG 200 in the nucleic acid amplification (21). Although PEG 200 increases the rate of chain exchange reactions between double-helix DNA and its homologous single-stranded DNA, no relevant studies have demonstrated that it can improve the reaction efficiency of RPA (22). We first verified the enhancement effect of PEG 200 on RPA according to the threshold cycle (*C_T_*) value change and ultimate fluorescence intensity, and the working concentration of 2.5% PEG 200 reduced the *C_T_* value from 18.302 to 15.649 (*P *< 0.01) and increased the final fluorescence intensity from 1,014,739 to 1,112,600 (*P *< 0.01), indicating that it significantly enhanced the amplification efficiency of the RPA (Fig. S6A and B). This enhancement may have occurred because the counterion association was alleviated during formation of the transitional intermediates in the sequential substitution pathway, thereby stabilizing the nucleation complex to accelerate hybridization (22). Next, we optimized the concentration of PEG 200, and 1% PEG 200 had the best enhancement effect on RPA, increasing the RPA amplification efficiency by 13% [calculated using the mean of ultimate fluorescence intensity (UFI): (UFI_1%_/UFI_0%_ − 1) × 100% (Fig. S7A and B)]. After optimization, the constructed HRP-catalyzed LFIA biosensor was used to further corroborate the RPA enhancement by PEG 200 through significant changes (*P* < 0.05) in the colorimetric signal of the test line (Fig. 3). For example, the signal intensity of the test line of the constructed LFIA biosensor increased significantly from 349.27 to 429.45 for *mcr-1*. The deepening color of the detection line of the constructed LFIA biosensor enabled better detection of the XDR genes. These results verified the effect of PEG 200, showing that it enhanced the RPA reaction, thus providing an important reference for developing RPA technology.

### Successfully prepared AuNPs and HRP-AuNP-antibody conjugate.

The detection probe is a critical factor in determining the performance of LFIA biosensors. Although AuNPs are commonly used to construct LFIA methods owing to their favorable biocompatibility and stability, the constructed assays usually exhibit low sensitivity (23). The HRP reaction catalyzed by its substrate (AEC) and H_2_O_2_ produced red nonsoluble products deposited on AuNPs, thus increasing the signal intensity of the test line on the lateral flow chromatography strips and ultimately enhancing the sensitivity of the whole reaction (17). Therefore, we used HRP-catalyzed enzymatic reactions to prepare a high-sensitivity LFIA and characterized the detection probes to ensure successful construction. Transmission electron microscopy (TEM) of the AuNPs before and after antibody encapsulation indicated that the synthetic AuNPs had a well-defined oval shape with a uniform diameter of ~27 nm, and the TEM image of the HRP-AuNP-antibody conjugate demonstrated a luminous ring around the AuNPs (Fig. S8A and B). The UV-Vis (UV-visible) spectrum revealed the plasmonic absorption band of unlabeled AuNPs with a maximum value of 526 nm and a slight shift of the surface plasmon absorption to 538 nm after labeling with HRP and antibiotin (Fig. S8C). These results indicated that antibiotin (the HRP conjugate) successfully modified the AuNPs.

### Optimization of PEG 200-enhanced RPA and HRP-catalyzed LFIA biosensor parameters.

To enhance the target detection sensitivity while ensuring detection specificity, we optimized the conditions of the PEG 200-enhanced RPA and HRP-catalyzed LFIA biosensors.

**(i) Unitary PEG 200-enhanced RPA.** We optimized the reaction temperature and time of the PEG 200-enhanced RPA by determining the reaction conditions with the highest ultimate fluorescence intensity using exo-RPA (Fig. S9). Because *tet*(X4) and *tet*(X6) required the same set of primers and probes, the optimal RPA reaction time and temperature for these two genes were the same. The final reaction time and reaction temperature were both determined (Table S3).

**(ii) HRP-catalyzed LFIA biosensor.** We further investigated the influence of the amount of antibiotin (HRP conjugate) used as a coating during preparation of the conjugate and the volume of the HRP-AuNP-antibody conjugate by comparing the analytical capabilities (peak area of the test line) of the HRP-catalyzed LFIA biosensor (Fig. S10 and S11) and determined the most suitable combination (Table S3). Subsequently, we optimized the volume of the enzymatic reaction substrate added dropwise to the strip. Because the HRP-catalyzed enzymatic reaction had similar enhancement effects on the colorimetric signal of the detection line of the test strips when detecting different targets, we applied the same volume of the enzymatic reaction substrate to all targets. The final volume of the enzymatic reaction substrate to be added dropwise to the test strip was 30 μL (Fig. S12).

### Sensitivity of the constructed cascade detection system reached within 10 copies/μL of the XDR genes.

**FIG 4:**
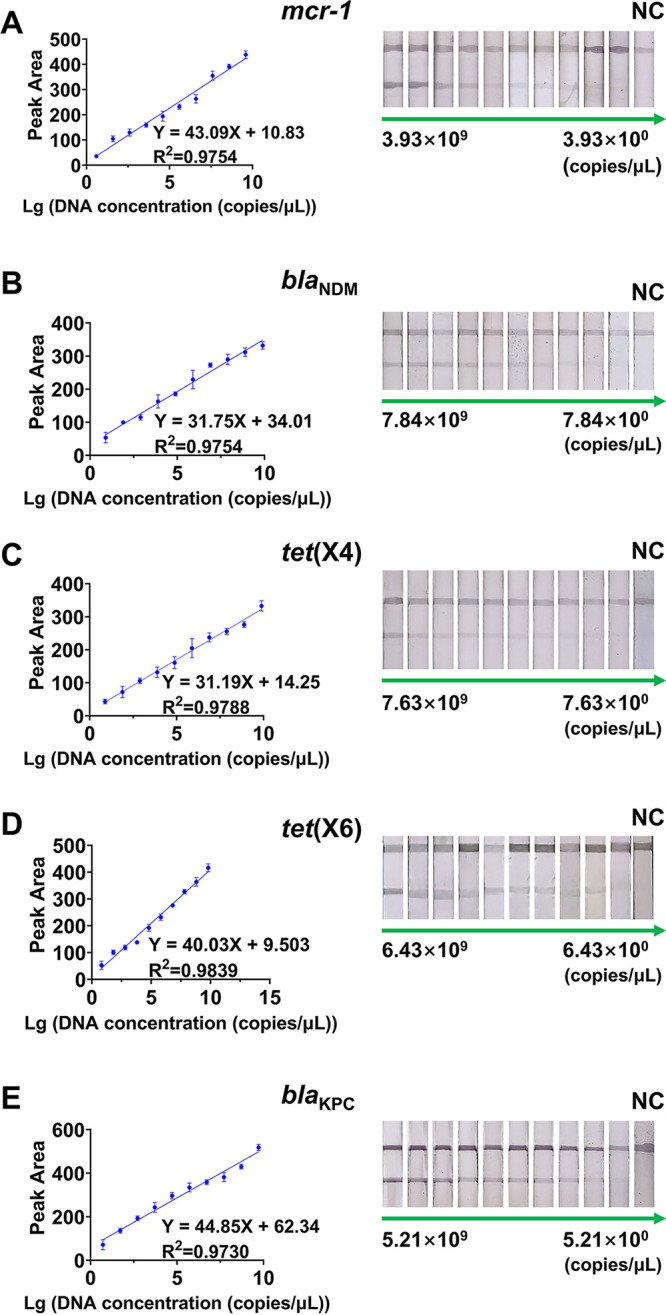
Sensitivity results of the cascade detection system for different XDR genes. Linear regression and typical image of *mcr-1* (A), *bla*_NDM_ (B), *tet*(X4) (C), *tet*(X6) (D), and *bla*_KPC_ (E) detection.

Sensitivity is an important index for evaluating the detection performance of the whole detection system. Under the optimized detection circumstances, the sensitivity of this cascade detection system was tested by determining different copy numbers (10^9^ to 10^0^ copies/μL) of the XDR genes. The copy number (copies per microliter) of double-stranded DNA was calculated as 6.02 × 10^23^ × 10^−9^ × DNA concentration (nanograms per microliter)/DNA length × 660. The intensity of the colorimetric signal of the test line decreased as the copies of the XDR genes decreased, and the negative control with nuclease-free water as the amplification template showed the expected negative results (Fig. 4). The visual limits of detection (vLODs) of the constructed cascade detection system for *mcr-1*, *bla*_NDM_, *tet*(X4), *tet*(X6), and *bla*_KPC_ were 3.93, 7.84, 7.63, 6.43, and 5.21 copies/μL (2.6, 4.4, 4.6, 3.7, and 3.0 fg), respectively, which were higher than those of loop-mediated isothermal amplifications and superior to the sensitivity of fluorescence quantitative PCR (qPCR) assays for detecting XDR genes (Table 1). More importantly, our cascade system performs bacterial DNA extraction using a modified Chelex-100 lysis method, which reduces the extraction time to less than 20 min compared to conventional commercial kits. Meanwhile, our constructed cascade system has the following advantages compared to qPCR and LAMP: (i) compared with qPCR, only a simple metal bath is needed to complete the nucleic acid amplification reaction without the use of expensive professional instruments, and (ii) compared with LAMP, only a 15-min reaction at 37°C is required for nucleic acid amplification, which requires more modest reaction conditions (LAMP requires a 40-min reaction at 65°C). In brief, the advantages of our cascade system over qPCR and LAMP are the ability to visually detect these XDR genes *in situ* without the need for specialized equipment and the shorter detection time and more modest reaction conditions required. The calculated limits of detection (cLODs) of the detection system for *mcr-1*, *bla*_NDM_, *tet*(X4), *tet*(X6), and *bla*_KPC_ were 2.36, 4.70, 4.58, 3.86, and 3.13 copies/μL, respectively. Considering that XDR genes are typically in multiple copies in bacteria and the copy number in wild-type bacteria is usually low, our system could commonly satisfy the demand (24).

**TABLE 1:** Comparison of the sensitivity of the constructed cascade detection system with those of established qPCR and LAMP assays

Target	This study (copies/μL)	qPCR (copies/μL)	LAMP (fg)	Reference(s)
*mcr-1*	3.93 (2.6 fg)	270	360	30, 31
*bla*_NDM_	7.84 (4.4 fg)	10	4.6	32, 33
*tet*(X4)	7.63 (4.6 fg)	8.42		34
*tet*(X6)	6.43 (3.7 fg)			
*bla*_KPC_	5.21 (3.0 fg)	10		32

### Favorable specificity of the cascade detection system.

**FIG 5:**
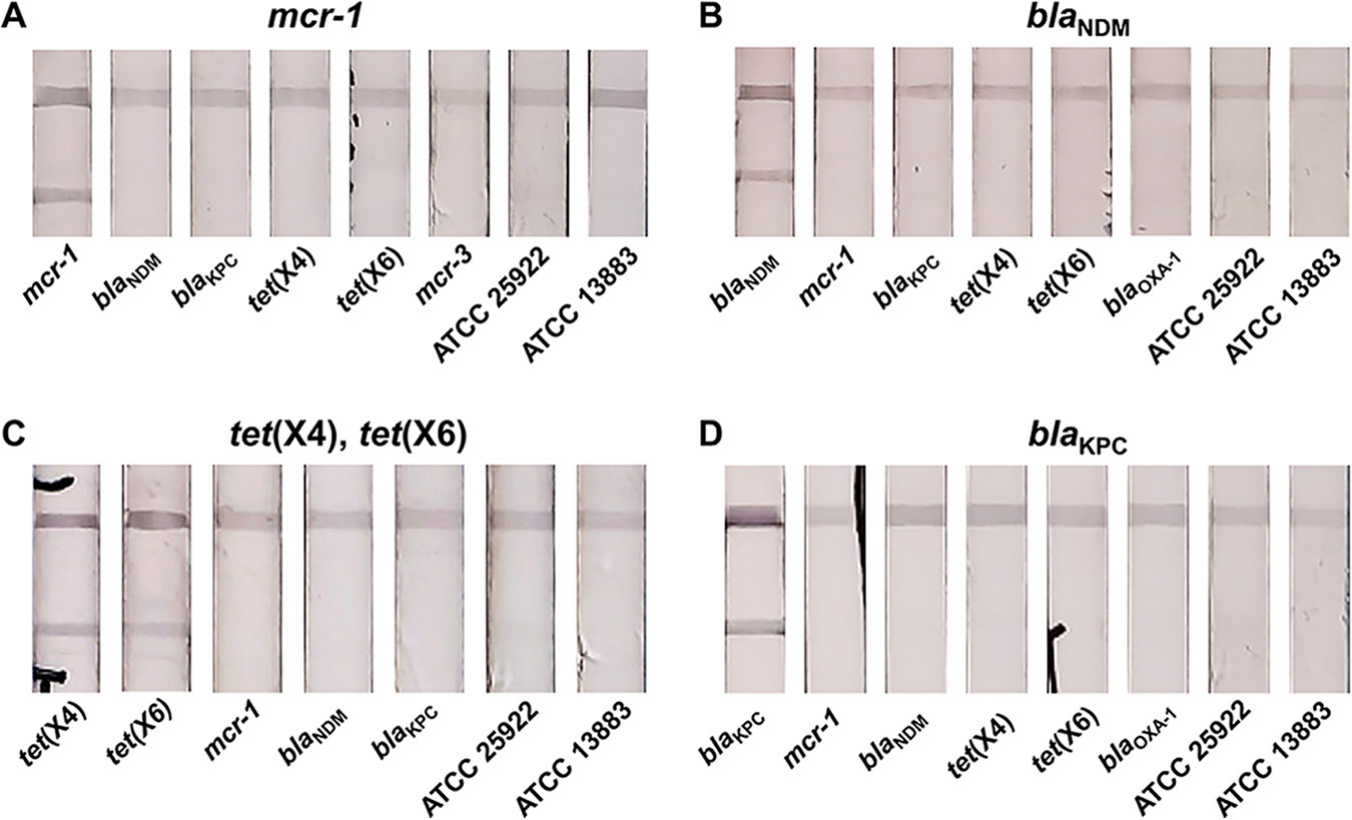
Specificity results of the cascade detection system for different interfering bacteria. Typical images of *mcr-1* (A), *bla*_NDM_ (B), *tet*(X4)/*tet*(X6) (C), and *bla*_KPC_ (D) detection. *mcr-1*, *bla*_NDM_, *tet*(X4), *tet*(X6), and *bla*_KPC_, constructed standard strains for *mcr-1*/*bla*_NDM_/*tet*(X4)/*tet*(X6)/*bla*_KPC_; *mcr-3*, *mcr-3*-positive E. coli; *bla*_OXA-1_, *bla*_OXA-1_-positive E. coli.

Specificity is a necessary indicator for evaluating the feasibility of the assay, and false-positive results should be avoided. To validate the specificity of the proposed detection system, we tested a series of *Enterobacteriaceae* bacterial samples, including the constructed standard *mcr-1*/*bla*_NDM_/*tet*(X4)/*tet*(X6)/*bla*_KPC_ strains, ATCC 25922, ATCC 13883, and *mcr-3*/*bla*_OXA-10_-positive strains collected in our lab. The test and control lines showed simultaneous color intensities only when the samples containing the corresponding target were detected. This optical signal appeared minimally in the other samples, indicating satisfactory specificity for the whole assay (Fig. 5). This favorable specificity is attributed to the designed combination of RPA primers and probes with high selectivity for the target genes (25).

### The cascade assay system showed consistency with PCR for actual sample detection.

Our goal is to enable rapid on-site detection of XDR genes in real specimens; thus, we verified the feasibility of the constructed method for detecting actual samples. To further verify its feasibility in practical applications, the cascade system and common PCR were applied simultaneously to detect *mcr-1*, *bla*_NDM_, *tet*(X4), *tet*(X6), and *bla*_KPC_ in 95 samples of *Enterobacteriaceae* under double-blind conditions. The sensitivity and specificity of the cascade assay system were obtained using the results of the common PCR assay as the standard. The sensitivities of the detection system for *mcr-1*, *bla*_NDM_, *tet*(X4)/*tet*(X6), and *bla*_KPC_ were 96.2% (95% confidence interval [CI], 80.4% to 99.9%), 95.0% (95% CI, 75.1% to 99.9%), 92.0% (95% CI, 74.0% to 99.0%), and 95.5% (95% CI, 77.2% to 99.9%), respectively, and the specificity was over 95% (Table 2). These results revealed a high degree of agreement between the two assays, indicating that this cascade system has great potential for application as a high-efficiency and credible alternative to PCR-based diagnostic tests for these XDR genes in *Enterobacteriaceae*.

**TABLE 2:** Sensitivity and specificity of the cascade detection system and PCR for detecting *mcr-1*, *bla*_NDM_, *tet*(X4)/*tet*(X6), and *bla*_KPC_^*a*^

XDR gene	No. of results				Sensitivity, %	Specificity, %
	PCR (+), cascade system (+)	PCR (+), cascade system (−)	PCR (−), cascade system (+)	PCR (−), cascade system (−)		
*mcr-1*	25	1	1	68	96.2 (25/25 + 1)	98.6 (68/68 + 1)
*bla*_NDM_	19	1	1	74	95.0 (19/19 + 1)	98.7 (74/74 + 1)
*tet*(X4)/*tet*(X6)	23	2	1	69	92.0 (23/23 + 2)	98.6 (69/69 + 1)
*bla*_KPC_	21	1	2	71	95.5 (21/21 + 1)	97.3 (71/71 + 2)

*^a^*Sensitivity, positive samples detected by both PCR and cascade system/total positive samples; specificity, negative samples detected by both PCR and cascade system/total negative samples.

In conclusion, we developed a rapid, *in situ*, visual cascade system for detecting the XDR genes *bla*_NDM_, *bla*_KPC_, *mcr-1*, *tet*(X4), and *tet*(X6) based on a unitary PEG 200-enhanced RPA combined with a modified Chelex-100 lysis method and an HRP-catalyzed LFIA biosensor. The method exhibited high sensitivity and satisfactory specificity, thus allowing better technical support for monitoring XDR genes and better service in clinical applications. Currently, clinical settings demand rapid detection of the XDR genes in samples to identify the antimicrobial agents to be used for fast treatment to achieve optimal therapeutic effects and to be able to minimize antimicrobial resistance. Therefore, further studies are needed to establish a method to detect the XDR genes directly from actual samples (e.g., urinary tract infection samples, etc.) without the preculture.

## MATERIALS AND METHODS

### Materials and chemicals.

Chloroauric acid tetrahydrate (HAuCl_4_·3H_2_O), trisodium citrate, hydrogen peroxide (H_2_O_2_), sodium chloride (NaCl), Chelex-100, Triton X-100, 3,3′,5,5′-3-amino-9-ethylcarbazole (AEC; the substrate for the enzymatic reaction catalyzed by HRP), polyethylene glycol (PEG) 200, bovine serum albumin (BSA), and phosphate-buffered saline (PBS; pH 7.4, 0.01 M) were purchased from Sigma Chemical Company (St. Louis, MO, USA). Tris-EDTA (TE) buffer solution and DNase/RNase-free water were obtained from Beijing Tiangen Biochemical Technology Co., Ltd. (China). The antidigoxin, antirhodamine, anti-FITC, anti-Cy3, and goat anti-rabbit IgG were acquired from Abcam Inc. (Cambridge, MA, USA). The antibiotin (HRP conjugate) was obtained from Cell Signaling Technology, Inc. (Boston, MA, USA). All RPA reagents used were purchased from Hangzhou Zhongce Biotechnology Co., Ltd. (China). Sample pads, nitrocellulose (NC) membranes (Sartorius CN140), absorbent pads, and backing cards were acquired from Kinbio Tech. Co., Ltd. (Shanghai, China).

### Bacterial strains.

The standard strains for *mcr-1*/*bla*_NDM_/*tet*(X4)/*tet*(X6) were constructed by cloning the *mcr-1*/*bla*_NDM_/*tet*(X4)/*tet*(X6) gene into pACYC184 and transforming it into Escherichia coli ATCC 25922. Moreover, the standard strains for *bla*_KPC_ were constructed by cloning the *bla*_KPC_ gene into pACYC184 and transforming it into Klebsiella pneumoniae ATCC 13883. In brief, the entire *bla*_NDM_, *bla*_KPC_, *mcr-1*, *tet*(X4), and *tet*(X6) open reading frames (ORFs) were amplified by PCR and cloned into pACYC184, resulting in recombinant plasmids pACYC184/*bla*_NDM_, pACYC184/*bla*_KPC_, pACYC184/*mcr-1*, pACYC184/*tet*(X4), and pACYC184/*tet*(X6), respectively. The plasmids (except for pACYC184/*bla*_KPC_) were then transformed into E. coli ATCC 25922 by electrotransformation. Also, pACYC184/*bla*_KPC_ was then transformed into K. pneumoniae ATCC 13883 in the same way. Ninety-five *Enterobacteriaceae* strains were collected from our lab and The Second Affiliated Hospital of Zhejiang University.

### Modification of conventional Chelex-100 lysis method.

Preparation of Chelex-100 lysis solution was carried out as follows: 2.5 g Chelex-100, 50 mL TE buffer, and 500 μL Triton X-100 were added in a 50-mL centrifuge tube and mixed well. The conventional Chelex-100 lysis method was performed with slight modifications based on previous studies (26). In order to achieve *in situ* extraction of bacterial DNA, we need to employ alternative methods to avoid the use of cumbersome high-speed centrifuges. Hence, the following steps were used instead of the original high-speed (13,000-rpm) centrifugation (see Fig. S1B in the supplemental material): (i) bacterial colonies were scraped from the plate into 1.5/2-mL centrifuge tubes using a 10-μL inoculation loop, and (ii) the bacterial solution was aspirated from the centrifuge tube using a 1-mL syringe and filtered through a 0.45/0.22-μm filter membrane. Since the actual effect of such a modification was not previously known, we added an additional step at the end: palmtop centrifugation for 1 min and retaining the supernatant. Bacterial DNA of the *mcr-1* standard strain was extracted using the conventional/modified Chelex-100 lysis method, respectively. Then, the quality of the extracted DNA (OD_260_/OD_280_, OD_260_/OD_230_) was measured using a NanoDrop 1000 UV-Vis spectrophotometer (Thermo Fisher Scientific, Waltham, MA, USA). Also, the concentration of double-stranded DNA in the extracted bacterial DNA was evaluated using Qubit (Thermo Fisher Scientific, Waltham, MA, USA). Bacterial DNA of 95 *Enterobacteriaceae* strains was extracted using a modified Chlex-100 lysis method with optimal results.

### Isothermal amplification.

**(i) Design of RPA primer and probe.** The RPA primer and probe of target XDR genes [*bla*_NDM_, *bla*_KPC_, *mcr-1*, *tet*(X4), and *tet*(X6)] were designed by PrimedRPA following TwistDx RPA kit guidelines (TwistDx, Cambridge, United Kingdom) (27). Afterward, NCBI Primer-BLAST (http://www.ncbi.nlm.nih.gov/tools/primer-blast/) and Primer premier 5.0 software (Biosoft International, Palo Alto, CA) were used to confirm the specificity of the primers and probes. All RPA primer and probe sequences used in this study are listed in Table S1 [*tet*(X4) and *tet*(X6) use the same set of RPA primers and probes].

**(ii) Enhancement of RPA by PEG 200.** In this study, the amplification efficiency of RPA was enhanced using PEG 200. PEG 200 is a kind of polyethylene glycol with a molecular weight of 200. It has been shown that PEG 200 is able to enhance the rate of strand exchange reaction between double-helix DNA and its homologous single-stranded DNA (22). Therefore, exo-RPA was used to validate the effect of PEG 200 on RPA amplification efficiency.

The exo-RPA was implemented in a 50-μL volume using an exo-RPA lyophilized kit purchased from Hangzhou Zhongce Biotechnology Co., Ltd. (China). It contains 13.5 μL buffer A (molecular crowding reagent), forward primer (10 μM, 2 μL), reverse primer-exo (10 μM, 2 μL), probe-exo (10 μM, 0.6 μL), 5 μL of the DNA template, magnesium acetate (MgOAc) (280 mM, 2.5 μL), PEG 200 in different working concentrations, and DNase/RNase-free water, making a final RPA reaction system of 50 μL. The *C_T_* value and ultimate fluorescence intensity were detected using the QuantStudio 7 Flex real-time PCR system (Applied Biosystems, Thermo Fisher Scientific) at 39°C for 15 min.

**(iii) Optimization of RPA reaction time and temperature for detection of XDR genes.** The exo-RPA was optimized in various circumstances. It was implemented at 39°C for 10, 15, 20, 25, and 30 min to determine the optimal reaction time. Moreover, to define the most suitable reaction temperature, the reaction was carried out at 35, 37, 39, 41, 43, and 45°C for the most appropriate time. The final reaction procedure was determined according to the *C_T_* value and ultimate fluorescence intensity.

### HRP-catalyzed lateral flow immunoassay biosensor.

**(i) Preparation of HRP-AuNP-antibody conjugate.** Gold nanoparticles (AuNPs) were synthesized according to the previous method (28). Briefly, 50 mL of an aqueous solution of 0.24 mM HAuCl_4_ was heated to boiling, and then 1.67 mL of 1% trisodium citrate was rapidly injected. The mixed solution was stirred for 30 min and then stored at 4°C before use.

The preparation of HRP-AuNP-antibody conjugate was performed according to the method of a previous study with slight modifications (29). A 20-μL aliquot of the antibiotin (HRP conjugate) was added to 1 mL AuNP solution (pH 8.0 to 8.5). After standing for 1 h, 50 μL of 20% BSA solution was added to block the excess reactivity of AuNPs and then incubated for another 1 h. Subsequently, the mixture was centrifuged at 11,000 rpm for 10 min at 4°C and the precipitate was retained. Finally, the precipitate was resuspended by adding 100 μL of the resuspension buffer (containing 0.5% [wt/vol] BSA, 0.5% [vol/vol] Tween 20, 2% [wt/vol] sucrose, and 50 mM Tris-HCl buffer) and stored at 4°C until use.

**(ii) Fabrication of the HRP-catalyzed lateral flow immunoassay strips.** The strips consisted of four parts: a sample pad, a nitrocellulose membrane, an absorption pad, and a backing card. The test line and control line were immobilized by dispensing antidigoxin antibody (or antirhodamine antibody, anti-FITC antibody, or anti-Cy3 antibody) solution (1.0 mg/mL) and goat anti-rabbit IgG solution (1.0 mg/mL) onto the nitrocellulose membrane at a rate of 1 μL/cm using the dispensing system purchased from Kinbio Tech. Co., Ltd. (Shanghai, China). Then, the NC membranes were desiccated at 37°C overnight and kept at 4°C. After that, the sample pad, nitrocellulose membrane, and absorption pad were assembled onto the backing card with an approximately 1- to 2-mm overlap to ensure that the solution could migrate smoothly. Finally, the constructed strips were cut at 3.0-mm individual width by the computer numerical control (CNC) high-speed cutting machine purchased from Kinbio Tech. Co., Ltd. (Shanghai, China), and stored at 4°C.

### Detection of the XDR genes [*bla*_NDM_, *bla*_KPC_, *mcr-1*, *tet*(X4), and *tet*(X6)].

The DNA template was acquired by the modified Chelex-100 lysis method. Subsequently, the PEG 200-reinforced nfo-RPA was implemented in a 50-μL volume using an nfo-RPA lyophilized kit purchased from Hangzhou Zhongce Biotechnology Co., Ltd. (China). It contains 12.5 μL buffer A, forward primer (250 nM, 2 μL), reverse primer-nfo (250 nM, 2 μL), probe-nfo (125 nM, 0.6 μL), 5 μL of the DNA template, MgOAc (280 mM, 2.5 μL), PEG 200 in the most suitable working concentration, and DNase/RNase-free water, making a final RPA reaction system of 50 μL. Next, the mixture was incubated in a metal heating block under the optimized RPA reaction conditions. Afterward, the prepared HRP-AuNP-antibody conjugate and 150 μL of running buffer (PBS) were mixed with 25 μL of the amplified product solution. Afterward, the mixture was loaded onto the sample pad of the constructed strips and caused to migrate upward by capillary forces. After 10 min of color development, the sample pad and absorbent pad were removed, and then the mixture of AEC and H_2_O_2_ was added dropwise to the nitrocellulose membrane. The lines were visually assessed within 5 min. Positive results revealed bands on both test and control lines, while negative results displayed a band on only the control line. The complete detection procedure could be completed within 1 h and without any bulky instrument. The calculated limit of detection (cLOD) was counted by averaging the signal intensities plus three times the standard deviation of the test line of the control group.

### Statistical methods.

The *t* test was used to compare discrepancies between the groups. The graph plotting and statistical analysis were performed with GraphPad Prism 7.
