# Supplementary material for: Rapid On-Site Detection of Extensively Drug-Resistant Genes in Enterobacteriaceae via Enhanced Recombinase Polymerase Amplification and Lateral Flow Biosensor
Source: Microbiol Spectr. 2022 Nov 29;10(6):e03344-22. doi: 10.1128/spectrum.03344-22 (PMC9769758; doi:10.1128/spectrum.03344-22)
Supplement: Supplemental file 1 — Tables S1 to S3 and Fig. S1 to S12. Download spectrum.03344-22-s0001.pdf, PDF file, 2.0 MB [file spectrum.03344-22-s0001.pdf]

Supporting Information

For

Rapid on-site detection of extensively drug-resistant genes in Enterobacteriaceae via enhanced recombinase polymerase amplification and lateral flow biosensor

Jin Tao[#], Dejun Liu[#], Jincheng Xiong, Leina Dou, Weishuai Zhai, Rong Zhang, Yang Wang, Jianzhong Shen*, Kai Wen*

Department of Veterinary Pharmacology and Toxicology, College of Veterinary Medicine, China Agricultural University, Beijing Key Laboratory of Detection Technology for Animal-Derived Food Safety, Beijing Laboratory for Food Quality and Safety, Beijing 100193, People's Republic of China

******Author to whom correspondence should be addressed*

E-mail: sjz@cau.edu.cn; wenkai@cau.edu.cn

Supplementary Table

Table S1. Sequences of the primers and probes

Oligonucleotide name	Sequence (5' -3')
mcr-1 RPA F	AGTATCTTGTGGCGTGATAATAATTCGGAC
mcr-1 RPA R exo	GTCATCTAAGCCAACGAGCATACCGACATC
mcr-1 RPA R nfo	/Biotin/-GTCATCTAAGCCAACGAGCATACCGACATC
mcr-1 RPA P exo	CCGCGACCAACAACGCCATCTGCAACACCAA(FAM-dT)C(THF)T(BHQ1-dT)ATAACGAATGCCGC(C3 spacer)
mcr-1 RPA P nfo	/Digoxin/-CCGCGACCAACAACGCCATCTGCAACACCA(THF)TCCTTATAACGAATGCCGC(C3 spacer)
bla$_{NDM}$ RPA F	TCTGGCAGCACACTTCCTATCTCGACATGC
bla$_{NDM}$ RPA R exo	CCGGCAGGTTGATCTCCTGCTTGATCCAGT
bla$_{NDM}$ RPA R nfo	/Biotin/-CCGGCAGGTTGATCTCCTGCTTGATCCAGT
bla$_{NDM}$ RPA P exo	ACGGTTTGATCGTCAGGGATGGCGGCCGCG(FAM-dT)GC(THF)GT(BHQ1-dT)GGTCGATACCGCC(C3 spacer)
bla$_{NDM}$ RPA P nfo	/TAMRA/-GATGGCGGCCGCGTGCTGTTGGTCGATACC(THF)CCTGGACCGATGACCAGAC(C3 spacer)
tet(X4), *tet*(X6) RPA F	CCGACAATATCAAGGCATCCATCAACCCGC
tet(X4), *tet*(X6) RPA R exo	CTACAAAGAACTGATTCGTGTGACATCATC
tet(X4), *tet*(X6) RPA R nfo	/Biotin/-CTACAAAGAACTGATTCGTGTGACATCATC
tet(X4), *tet*(X6) RPA P exo	GAGGCATCAAATGAGCAGCATCTCCAATCA(FAM-dT)C(THF)T(BHQ1-dT)ATGGGTAATGGACGC(C3 spacer)
tet(X4), *tet*(X6) RPA P nfo	/FITC/-GAGGCATCAAATGAGCAGCATCTCCAATCA(THF)CGTTATGGGTAATGGACGC(C3 spacer)
bla$_{KPC}$ RPA F	AACCATTCGCTAAACTCGAACAGGACTTTG
bla$_{KPC}$ RPA R exo	GAAAGCCCTTGAATGAGCTGCACAGTGGGA
bla$_{KPC}$ RPA R nfo	/Biotin/-GAAAGCCCTTGAATGAGCTGCACAGTGGGA
bla$_{KPC}$ RPA P exo	ACGCGATGGATACCGGCTCAGGCGCAACTG(FAM-dT)A(THF)G(BHQ1-dT)TACCGCGCTGAGGAG(C3 spacer)
bla$_{KPC}$ RPA P nfo	/Cy3/-ACGCGATGGATACCGGCTCAGGCGCAACTG(THF)AAGTTACCGCGCTGAGGAG(C3 spacer)

Table S2. Validation of DNA extraction by modified Chelex-100 lysis method using strains collected from environmental and clinical sources

Sample ID	Species	Double-stranded DNA (ng/µL)	OD_{260}/OD_{280}	OD_{260}/OD_{230}
1	*E. coli*	39.2	1.49	0.30
2	*E. coli*	45.4	1.48	0.35
3	*E. coli*	16.1	1.42	0.59
4	*K. pneumoniae*	37.0	1.45	0.68
5	*E. coli*	42.4	1.56	0.67
6	*E. coli*	43.8	1.52	0.72
7	*E. coli*	44.6	1.53	0.45
8	*E. coli*	26.0	1.44	0.52
9	*E. coli*	44.6	1.35	0.50
10	*E. coli*	41.8	1.32	0.50
11	*E. coli*	42.4	1.37	0.42
12	*E. coli*	48.4	1.40	0.46
13	*E. coli*	45.4	1.44	0.57
14	*E. coli*	48.2	1.52	0.78
15	*E. coli*	46.0	1.64	0.31
16	*E. coli*	44.2	1.62	0.35
17	*E. coli*	42.0	1.72	0.42
18	*E. coli*	43.4	1.60	0.54
19	*E. coli*	45.6	1.50	0.41
20	*E. coli*	48.0	1.51	0.32
21	*E. coli*	43.6	1.42	0.78
22	*E. coli*	49.8	1.45	0.67
23	*E. coli*	46.6	1.57	0.65
24	*E. coli*	49.2	1.67	0.58
25	*E. coli*	56.0	1.62	0.54
26	*K. pneumoniae*	42.4	1.58	0.52
27	*E. coli*	48.4	1.54	0.61
28	*E. coli*	32.8	1.32	0.72
29	*E. coli*	58.4	1.41	0.61
30	*E. coli*	50.6	1.40	0.60
31	*E. coli*	51.8	1.39	0.49
32	*K. pneumoniae*	42.2	1.35	0.48
33	*E. coli*	53.2	1.28	0.41
34	*E. coli*	48.2	1.19	0.52
35	*E. coli*	50.6	1.25	0.49
36	*E. coli*	51.8	1.31	0.43
37	*E. coli*	55.4	1.34	0.44
38	*E. coli*	55.2	1.56	0.63
39	*E. coli*	47.8	1.71	0.62
40	*E. coli*	55.4	1.72	0.71

41	*E. coli*	56.4	1.60	0.32
42	*E. coli*	54.8	1.61	0.45
43	*E. coli*	44.2	1.62	0.46
44	*E. coli*	50.8	1.65	0.52
45	*K. pneumoniae*	42.4	1.35	0.85
46	*K. pneumoniae*	31.0	1.37	0.82
47	*E. coli*	56.0	1.45	0.72
48	*K. pneumoniae*	45.8	1.43	0.75
49	*E. coli*	31.8	1.42	0.85
50	*K. pneumoniae*	51.4	1.41	0.80
51	*E. coli*	47.8	1.52	0.70
52	*E. coli*	37.2	1.57	0.74
53	*E. coli*	39.2	1.58	0.64
54	*E. coli*	39.2	1.61	0.87
55	*E. coli*	50.0	1.63	0.76
56	*E. coli*	41.0	1.32	0.85
57	*E. coli*	55.8	1.37	0.87
58	*E. coli*	44.2	1.45	0.74
59	*E. coli*	51.4	1.53	0.70
60	*E. coli*	52.6	1.62	0.68
61	*K. pneumoniae*	41.6	1.44	0.74
62	*E. coli*	50.2	1.35	0.84
63	*E. coli*	51.8	1.32	0.82
64	*E. coli*	50.2	1.37	0.85
65	*E. coli*	43.0	1.40	0.81
66	*K. pneumoniae*	30.6	1.44	0.74
67	*E. coli*	42.4	1.45	0.86
68	*K. pneumoniae*	42.0	1.38	0.83
69	*K. pneumoniae*	30.6	1.45	0.59
70	*K. pneumoniae*	37.8	1.42	0.68
71	*K. pneumoniae*	31.6	1.51	0.72
72	*K. pneumoniae*	33.4	1.54	0.73
73	*K. pneumoniae*	34.2	1.57	0.54
74	*K. pneumoniae*	42.0	1.40	0.61
75	*K. pneumoniae*	44.6	1.44	0.60
76	*K. pneumoniae*	35.4	1.35	0.52
77	*K. pneumoniae*	31.6	1.32	0.51
78	*K. pneumoniae*	41.4	1.39	0.43
79	*K. pneumoniae*	33.8	1.74	0.42
80	*K. pneumoniae*	35.4	1.72	0.41
81	*K. pneumoniae*	43.6	1.65	0.39
82	*K. pneumoniae*	39.8	1.62	0.35
83	*K. pneumoniae*	41.2	1.53	0.40
84	*K. pneumoniae*	51.6	1.42	0.37

85	*K. pneumoniae*	45.8	1.40	0.41
86	*K. pneumoniae*	40.0	1.37	0.35
87	*K. pneumoniae*	41.8	1.39	0.38
88	*K. pneumoniae*	37.8	1.32	0.38
89	*K. pneumoniae*	36.2	1.57	0.40
90	*K. pneumoniae*	39.4	1.46	0.45
91	*P.mirabilis*	29.8	1.43	0.48
92	*P.mirabilis*	19.5	1.45	0.54
93	*E. coli*	36.8	1.39	0.85
94	*E. coli*	45.4	1.68	0.82
95	*E. coli*	56.6	1.71	0.59

Table S3 Detection conditions for RPA amplification and HRP-catalyzed lateral flow immunoassay biosensor of *mcr-1*, *bla*$_{NDM}$, *tet*(X4)/*tet*(X6), *bla*$_{KPC}$

**Target**	**RPA reaction time (min)**	**RPA reaction temperature (°C)**	**Amount of anti-biotin (HRP conjugate) (μg)**	**Volume of HRP-AuNPs-antibody conjugate (μL)**	**Volume of enzymatic reaction substrate (μL)**
mcr-1	15	41	5	5	30
bla$_{NDM}$	15	43	7	3	30
tet(X4)/*tet*(X6)	15	41	3	1.5	30
bla$_{KPC}$	15	41	5	4	30

Supplementary figures

Fig. S1

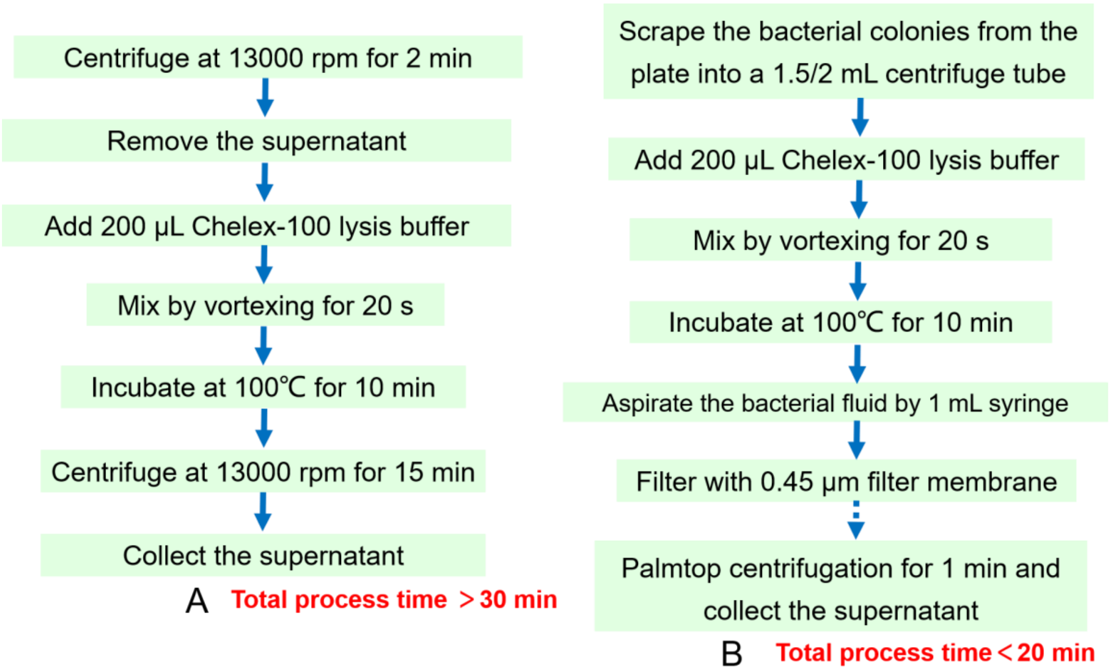

Fig. S1 Steps of the conventional and modified Chelex-100 lysis method: (A) conventional method; (B) modified method.

Fig. S2

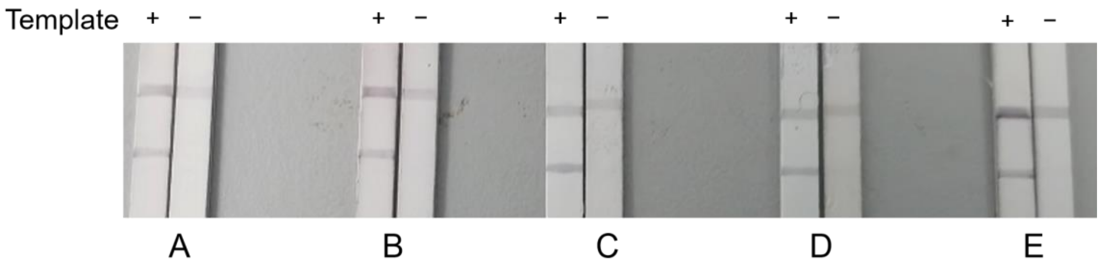

Fig. S2 Validation of nfo-RPA primers and probe for *mcr-1*, *bla*$_{\mathbf{NDM}}$, *tet*(X4)/*tet*(X6), *bla*$_{\mathbf{KPC}}$ with/without amplification template: (A) *mcr-1*; (B) *bla*$_{\mathbf{NDM}}$; (C) *tet*(X4); (D) *tet*(X6); (E) *bla*$_{\mathbf{KPC}}$.

Fig. S3

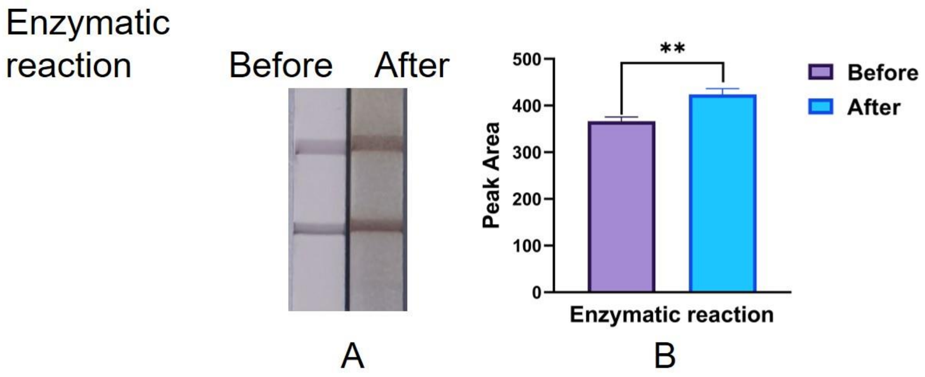

Fig. S3 Validation of HRP-catalyzed enzymatic reaction-enhanced lateral flow biosensor for detection signal enhancement: (A) before and after enzymatic reaction; (B) peak area of the test line.

Fig. S4

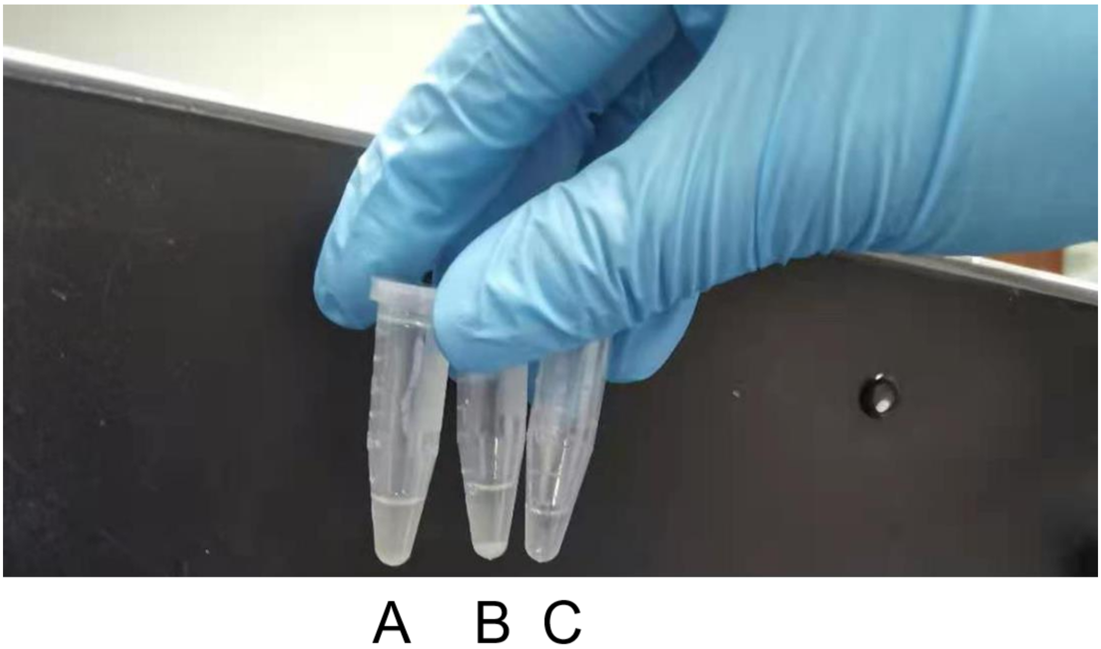

Fig. S4 Comparison of the performance of Chelex-100 lysis method before/after modification: (A) untreated; (B) before modification; (C) after modification.

Fig. S5

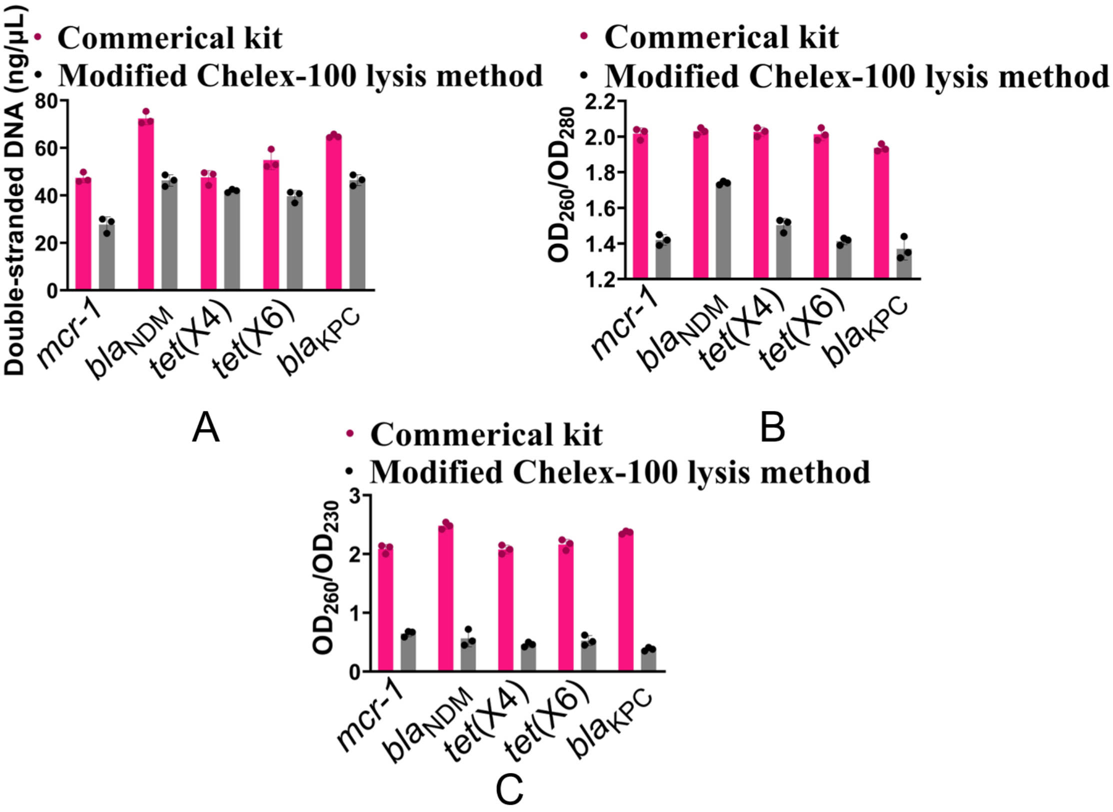

Fig. S5 Validation of the effect of DNA extraction by modified Chelex-100 lysis method using constructed standard strains: (A) concentration of double-stranded DNA; (B) value of OD_{260}/OD_{280}; (C) value of OD_{260}/OD_{230}.

Fig. S6

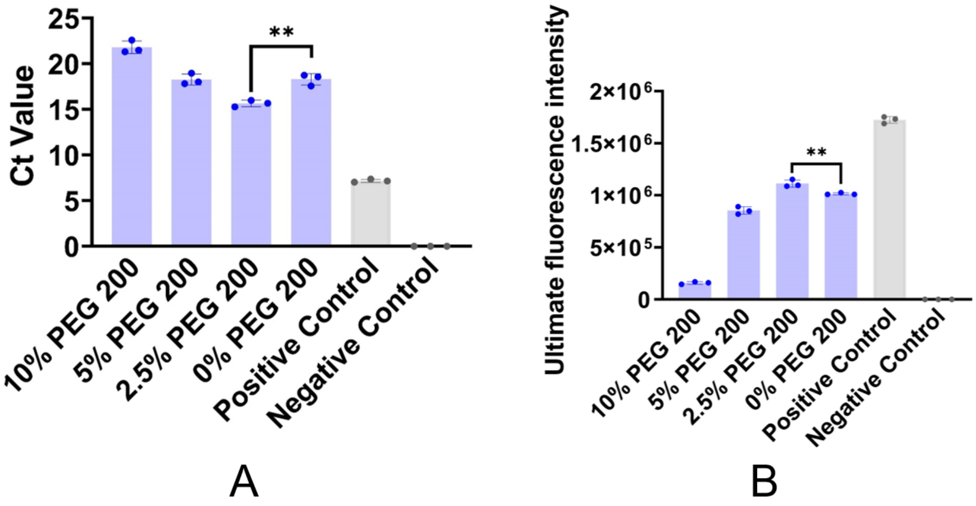

Fig. S6 Validation of the enhancement of RPA amplification efficiency by PEG 200: (A) Ct value; (B) ultimate fluorescence intensity.

Fig. S7

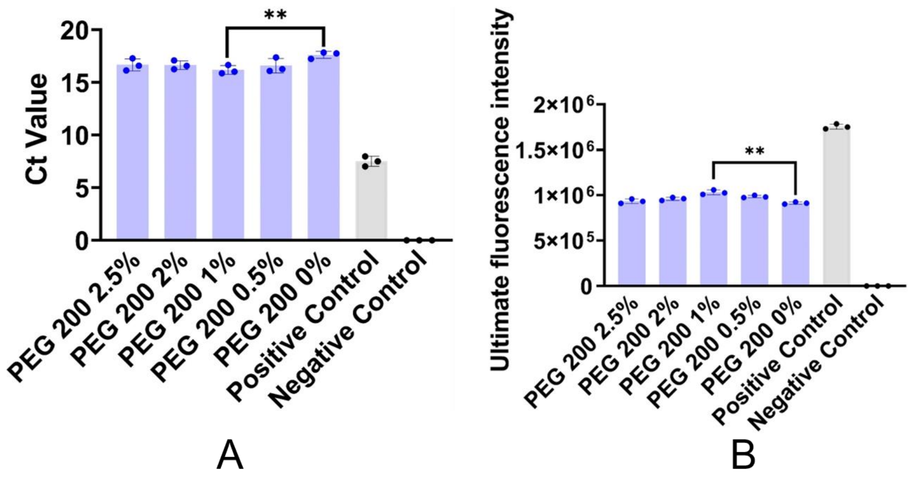

Fig. S7 Optimization of the working concentration of PEG 200: (A) Ct value; (B) ultimate fluorescence intensity.

Fig. S8

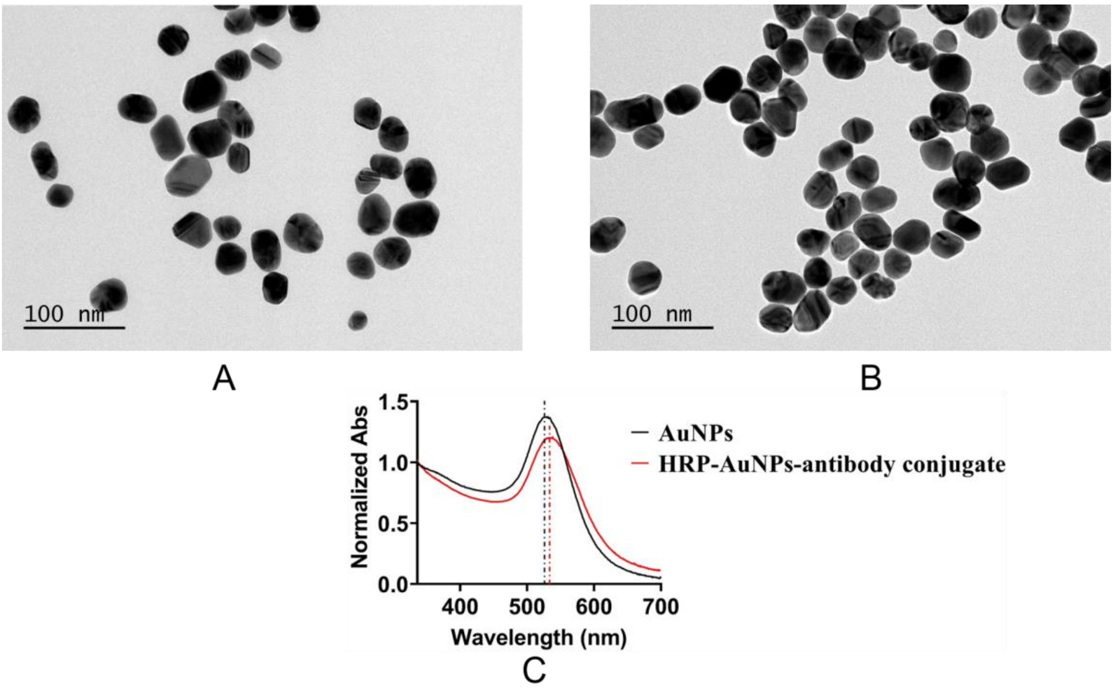

Fig. S8 Characterizations of AuNPs and HRP-AuNPs-antibody conjugate using TEM and UV-vis spectrum: (A) TEM image of AuNPs; (B) TEM image of HRP-AuNPs-antibody conjugate; (C) UV-vis spectrum of AuNPs and HRP-AuNPs-antibody conjugate.

Fig. S9

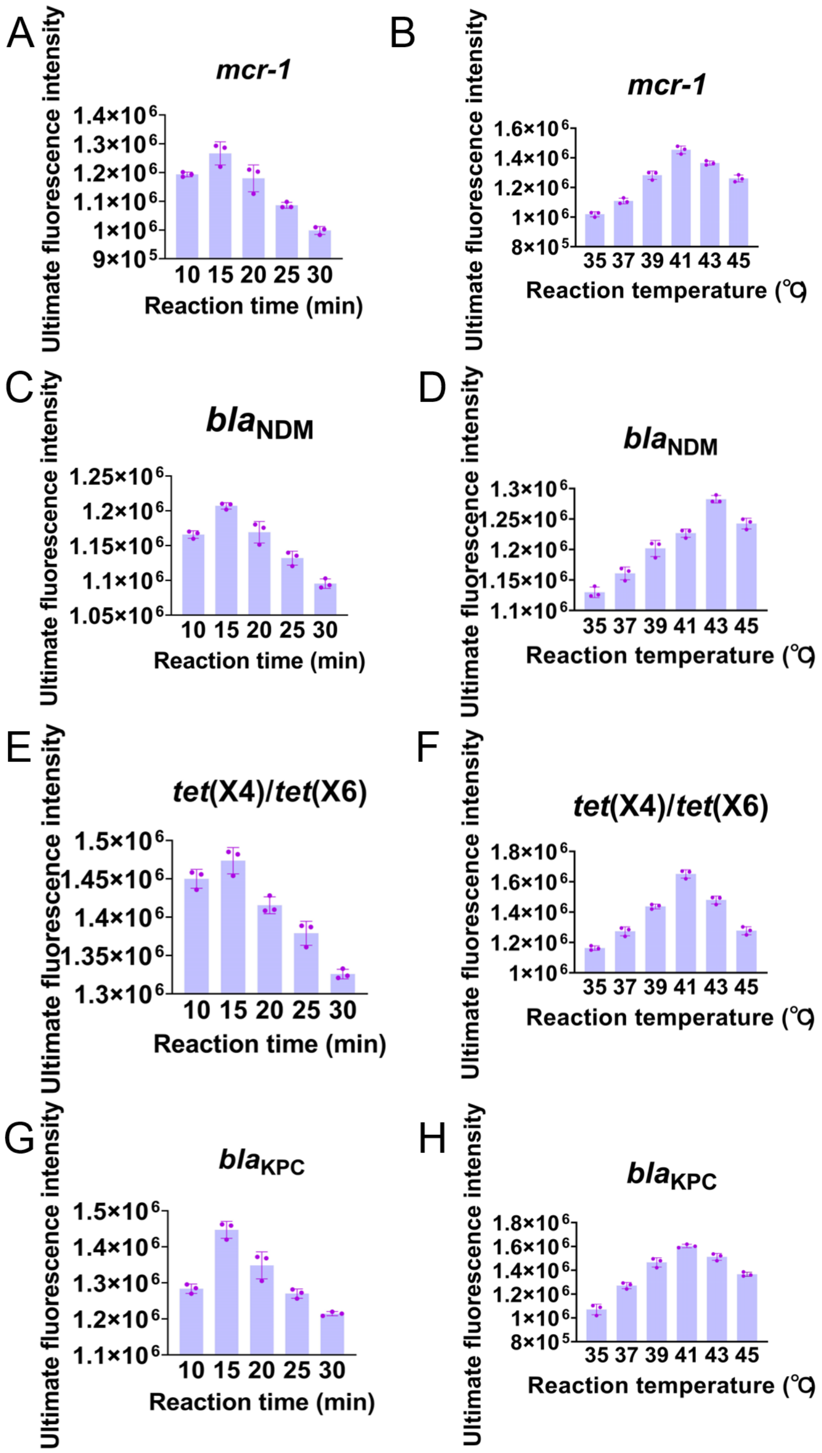

Fig. S9 Optimization of RPA reaction conditions: (A), (B) RPA reaction time and temperature of *mcr-1*; (C), (D) RPA reaction time and temperature of *bla*$_{\text{NDM}}$; (E), (F) RPA reaction time and temperature of *tet*(X4)/*tet*(X6); (G), (H) RPA reaction time and temperature of *bla*$_{\text{KPC}}$.

Fig. S10

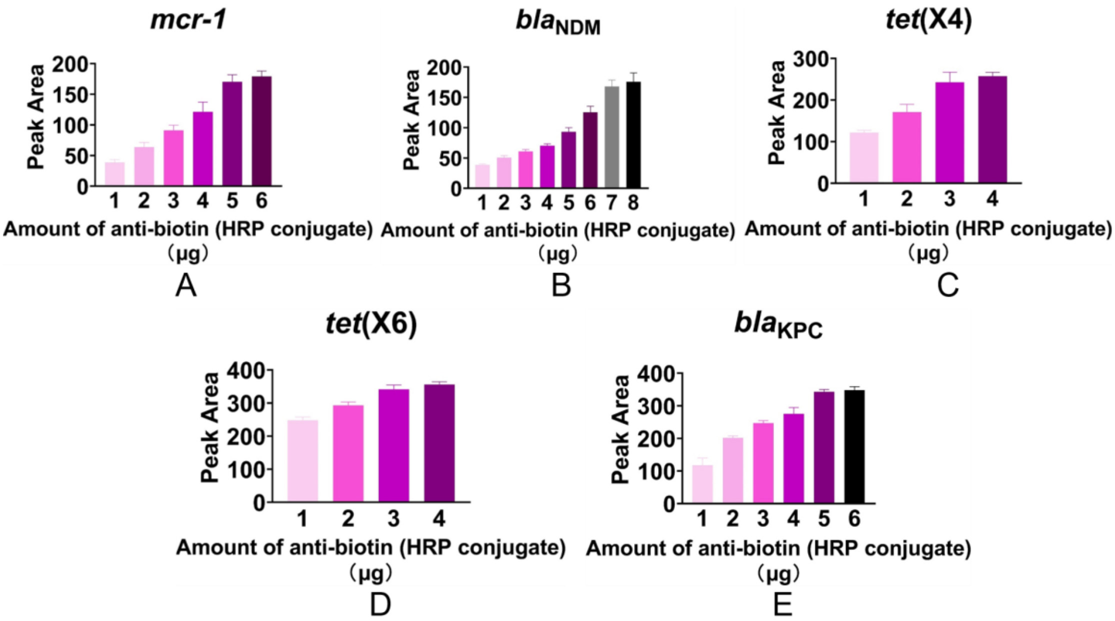

Fig. S10 Optimization of the amount of anti-biotin (HRP conjugate): (A) *mcr-1*; (B) *bla*$_{\text{NDM}}$; (C) *tet*(X4); (D) *tet*(X6); (E) *bla*$_{\text{KPC}}$.

Fig. S11

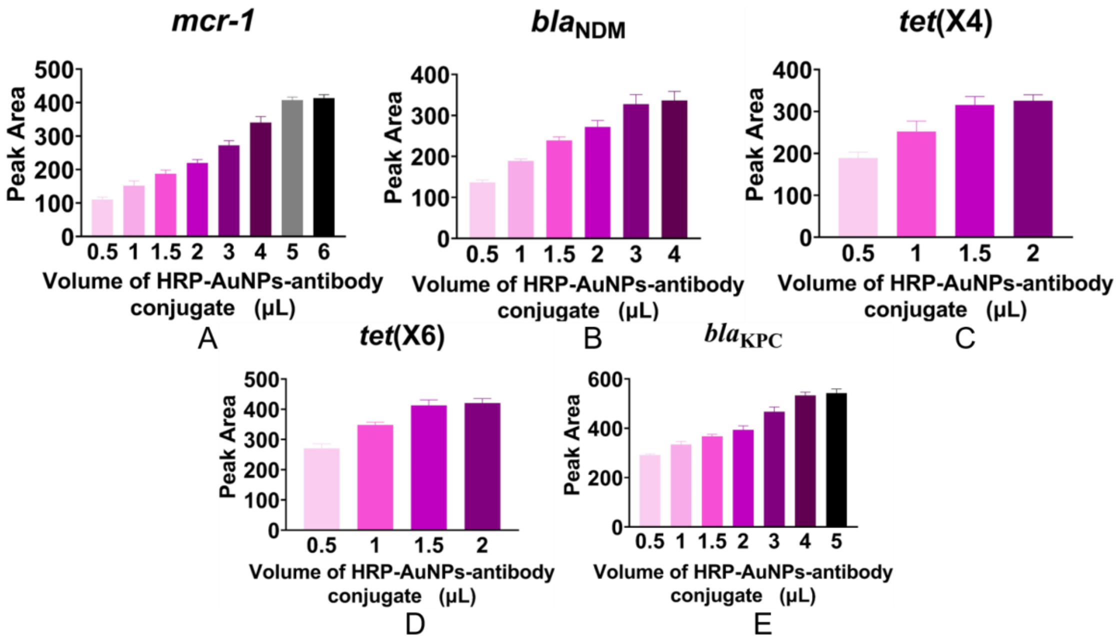

Fig. S11 Optimization of the volume of HRP-AuNPs-antibody conjugate: (A) *mcr-1*; (B) *bla*$_{\text{NDM}}$; (C) *tet*(X4); (D) *tet*(X6); (E) *bla*$_{\text{KPC}}$.

Fig. S12

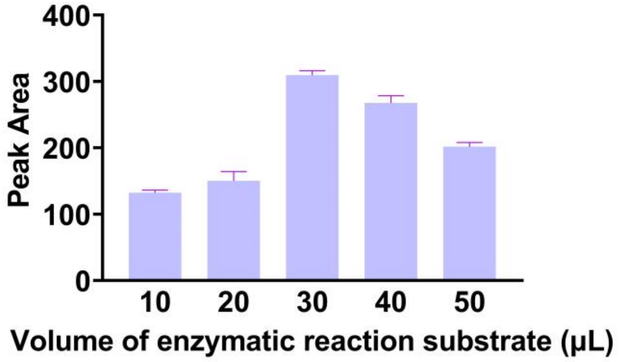

Fig. S12 Optimization of the volume of enzymatic reaction substrate.
